# Longitudinal characterization of multispecies microbial populations recovered from spaceflight potable water

**DOI:** 10.1038/s41522-021-00240-5

**Published:** 2021-09-06

**Authors:** Jiseon Yang, Jennifer Barrila, C. Mark Ott, Olivia King, Rebekah Bruce, Robert J. C. McLean, Cheryl A. Nickerson

**Affiliations:** 1 Biodesign Center for Immunotherapy, Vaccines and Virotherapy, Biodesign Institute, Arizona State University, Tempe, AZ USA; 2 Biodesign Center for Fundamental and Applied Microbiomics, Biodesign Institute, Arizona State University, Tempe, AZ USA; 3 Biomedical Research and Environmental Sciences Division, NASA Johnson Space Center, Houston, TX USA; 4 Department of Infectious Disease, Imperial College London, London, UK; 5 Department of Biology, Texas State University, San Marcos, TX USA; 6 School of Life Sciences, Arizona State University, Tempe, AZ USA

**Keywords:** Microbiome, Biofilms

## Abstract

While sequencing technologies have revolutionized our knowledge of microbial diversity, little is known about the dynamic emergent phenotypes that arise within the context of mixed-species populations, which are not fully predicted using sequencing technologies alone. The International Space Station (ISS) is an isolated, closed human habitat that can be harnessed for cross-sectional and longitudinal functional microbiome studies. Using NASA-archived microbial isolates collected from the ISS potable water system over several years, we profiled five phenotypes: antibiotic resistance, metabolism, hemolysis, and biofilm structure/composition of individual or multispecies communities, which represent characteristics that could negatively impact astronaut health and life-support systems. Data revealed a temporal dependence on interactive behaviors, suggesting possible microbial adaptation over time within the ecosystem. This study represents one of the most extensive phenotypic characterization of ISS potable water microbiota with implications for microbial risk assessments of water systems in built environments in space and on Earth.

## Introduction

Technological advances in recent years have enabled unprecedented insight into potable water microbiota using culture-independent methods (e.g., next-generation sequencing), revealing the vastly underestimated diversity in these communities. However, dynamic microbial interactions and phenotypes cannot be predicted based on sequencing information alone. Control of microbes in complex microbial ecosystems remains an important challenge, particularly with regard to the prevention and control of biofilms^1–5^. Changes that occur to the microbial ecosystem and the water-system integrity or function can impact the balance and population dynamics of resident microbiota, resulting in the release of pathogens or toxic materials to users and the environment. In addition to the health risks associated with microbial contaminants and their by-products, biofouling can cause materials degradation and systems’ failure^6^.

Microbial observatories have been established in a wide range of habitats to facilitate prolonged investigations into microbial communities^7^. The International Space Station (ISS) has been designated as a microbial observatory to enable research into microbial responses to the unique environment of spaceflight^8^. The ISS is the largest human-engineered structure in space and has harbored over two hundred international crew members since 2000. The ISS serves as a valuable observatory to study microbial populations, especially since human and microbial influx is well-controlled and limited compared with most built environments. Sustainable life support is provided to the crew through regenerated air and water by the Environmental Control and Life Support System (ECLSS)^9^. To ensure crew health and safety, NASA has diligently preserved ISS air, water, and surface microbial samples for years^10,11^. An important part of ECLSS is the water-recovery system (WRS), which generates potable water from recycled urine, wastewater, and condensation using multiple mechanical and chemical purification processes^9,12–14^. Even with these harsh treatments, including distillation, filtrations, catalytic oxidation, and iodine treatment^13–16^, a variety of microbes are routinely isolated from ISS potable water, and previous in-flight water analyses indicated that bacterial concentrations have exceeded the current ISS specifications of 50 CFU/mL for all potable water samples^17–19^. Sources of contamination are reported to be mostly derived from the environmental flora entrenched in the water system itself^18^. Historical monitoring of the NASA ISS potable water system indicates a stable microbial population consisting of relatively few microbial species^11^.

The presence of microbes in ISS potable water is not surprising, given their presence in drinking water on Earth. For example, *Ralstonia, Burkholderia, Sphingomonas*, and *Rhizobiales* recovered aboard the ISS are also commonly found in terrestrial potable or purified water systems^3,10,11,20–24^. Although ISS water contains the same types of microbes found in purified or potable water systems on Earth, there are several causes for concern. First, microbial ecosystems aboard the ISS are consistently exposed to the chronic stress of the microgravity environment, which is known to cause unexpected microbial responses that can impact human health (including enhanced virulence, biofilm formation, and antimicrobial resistance)^8,25–35^. Microgravity may present a selective pressure that could shape the adaptation/evolution of microorganisms. Second, many ISS potable water isolates are known to be strong biofilm formers^23,24,36,37^. This is of concern, as biofilms can be detrimental to the integrity and function of materials, inherently difficult to remove due to increased resistance to disinfectants and other antimicrobials, and present health risks to the crew^38^. Moreover, biofilm formation has been observed to increase in some microorganisms during spaceflight^28,31^. Finally, astronauts exhibit aspects of immune dysfunction during spaceflight and thus may be at increased risk for infection by both obligate and opportunistic pathogens^11,39,40^.

Previous studies using both culture-dependent and -independent approaches have reported findings regarding microbial composition and concentrations in ISS potable water^18,41–43^. To better understand the factors involved in regulating how these ISS microbial populations can survive and maintain their ecosystem, it is important to augment these studies with functional phenotypic assessments of both individual and mixed cultures. As alterations in microbial characteristics can impact the integrity of onboard life-support systems and astronaut health, we determined whether ISS bacterial water isolates changed their interactions and community properties over time, which could potentially impact material integrity and infectious disease risks. In this study, we examined sixteen ISS potable water isolates collected over several years for a variety of phenotypes, including biofilm formation and susceptibility to various antimicrobials. Our results indicated that the microbes were resistant to multiple drugs, and combinations of antibiotics were necessary for complete elimination. When we investigated interactions between species, we found that interactive behaviors of some of the isolates appeared to depend on whether they were recovered during the same year or different years, suggesting that for these select organisms, a common history of coexistence may influence their interactions. In agreement with recent findings^43^, we also observed *Burkholderia* species subpopulations that displayed hemolytic activity, which highlights a key phenotype that could adversely impact human health (particularly for individuals with blunted immune systems) and should thus be further explored for prevalence and associated risk in drinking water both aboard the ISS and in public water sources on Earth.

## Results

### Population analysis of archived ISS potable water microbiota

**Fig. 1:**
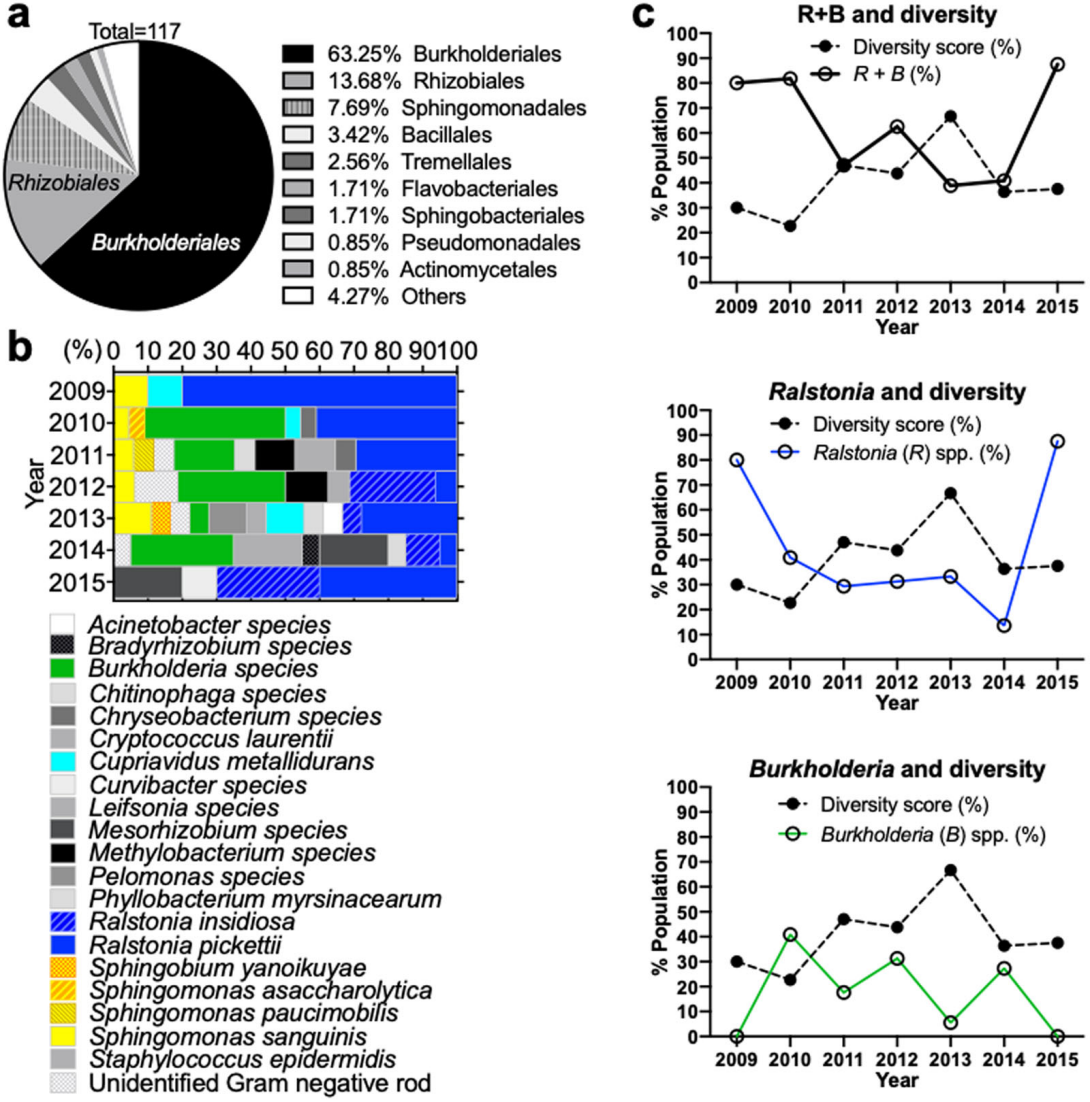
Population analysis of archived ISS potable water microbiota. **a** Relative abundance of the most common bacterial orders for archived ISS potable water isolates. **b** Percent population of different species of the ISS potable water isolates archived in 2009–2015. **c** Correlation between dominant species and microbial diversity in the ISS potable water. Diversity score was calculated as % = (the # of isolates of a species)/(total # of recovered microbes) × 100. The *y*-axis indicates the percentage of diversity (diversity score) or the indicated species.

To investigate dynamic changes in microbial species in the ISS Water Recovery System since its installation in 2008^13^, we analyzed a NASA-provided list of ISS potable water isolates preserved between 2009 and 2015^44^. Seventeen different microbial genera and five unidentified Gram-negative rods were represented from the list, which included a total of 117 potable water isolates. All microbial genera are listed in Supplementary Table 1. Our analysis showed Burkholderiales, which includes the genera *Burkholderia* (mostly *Burkholderia cepacia* complex, Bcc), *Ralstonia*, and *Cupriavidus*, to be the predominant order comprising 63.25% of the total ISS water isolates, and *Ralstonia pickettii* to be the major species of the order (Fig. 1a–b**)**. We further calculated the microbial diversity for each year by dividing the number of different species isolated in a year by the total number of isolates recovered in the year **(**Supplementary Table 2**)**. We found that changes in the microbial species diversity inversely correlated with both *Burkholderia* and *Ralstonia* spp. over time, with the relationship more strongly associated with the former than the latter (Fig. 1c). The composition of microbial species was similar as the same species were frequently isolated each year (Fig. 1b). To investigate how microbial communities in the ISS potable water system may have changed over time, we selected different species of ISS water microbiota isolated in early (2009), middle (2012), and late (2014) years for further phenotypic analysis, to better understand targeted population shifts that could negatively impact astronaut health and life-support systems (Table 1).

**Table 1:** Strains used in this study. ISS potable water isolates were recovered from the US system. The identifier is provided by NASA. The *Burkholderia cepacia* complex (Bcc) strains, indicated with *, were recently further re-identified^43^.

	Strain (year-#)	Identifier (by NASA)	Sample Collection Date	Microbial Identification
**2009**	2009-1	092160068-1	5/4/09	*Ralstonia pickettii*
	2009-2	092950004-2	9/22/09	*Sphingomonas sanguinis*
	2009-3	092570005-1	8/4/09	*Cupriavidus metallidurans*
**2012**	2012-1*	121850007-1	5/15/12	Bcc Burkholderia multivorans* *→* *B. cepacia s37*
	2012-2	121850007-2	5/15/12	*Ralstonia pickettii*
	2012-3	121560039-2	5/15/12	*Ralstonia insidiosa*
	2012-4	121850020-1	5/15/12	*Sphingomonas sanguinis*
	2012-5	121850020-3	5/15/12	*Methylobacterium species*
	2012-6	130770013-1	12/12/12	*Ralstonia insidiosa*
**2014**	2014-1*	140710050-1	2/3/14	Bcc Burkholderia multivorans* *→* *B. contaminans s52*
	2014-2*	140710038-1	2/3/14	Bcc Burkholderia species* *→* *B. contaminans s54*
	2014-3	140710038-2	2/3/14	*Ralstonia pickettii*
	2014-4	142550027-2	8/6/14	*Ralstonia insidiosa*
	2014-5	142550019-1	6/16/14	*Bradyrhizobium species*
	2014-6	142550021-2	8/6/14	*Mesorhizobium species*
	2014-7	150790064-1	12/31/14	*Staphylococcus epidermidis*

### Antimicrobial resistance of the ISS potable water isolates

**Fig. 2:**
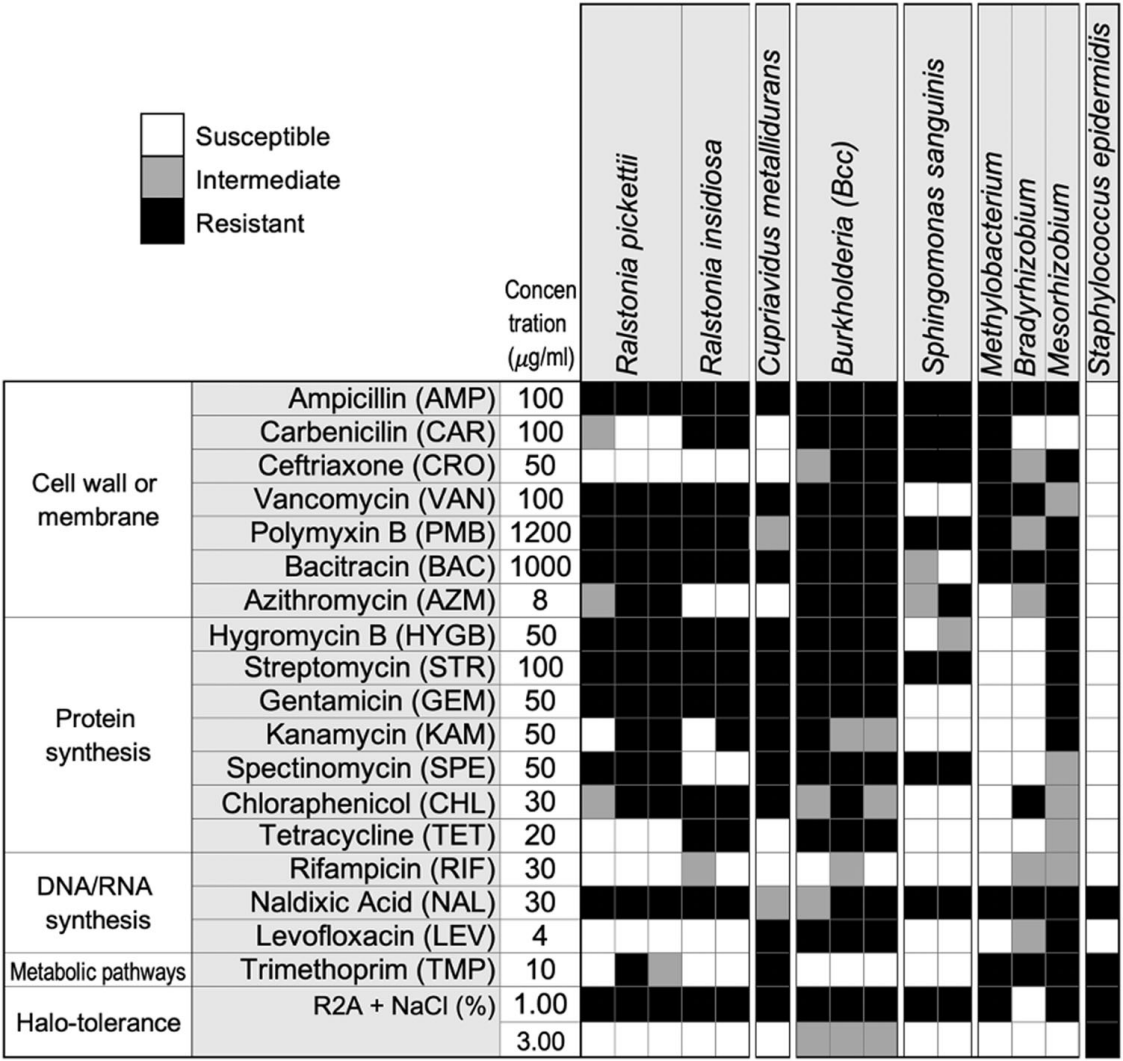
Antimicrobial susceptibility of the ISS potable water isolates, pure cultures. Summary of antimicrobial susceptibility of ISS potable water isolates is shown. Shading in gray scale from black to white indicates responses ranging from resistant to sensitive, respectively. The data from representative concentrations for selected antimicrobials, commonly used for Gram-negative bacteria, are shown. The complete results are in Supplementary Dataset 1.

We profiled each of the individual bacterial isolates for their susceptibility to twenty antimicrobial compounds, as well as their tolerance to salt (halotolerance) (Fig. 2). The complete list of antimicrobial compounds, tested concentrations, and test results is shown in Supplementary Dataset 1. All strains were resistant to multiple drugs at the concentrations tested for this study, most of which agreed with known–intrinsic drug resistance for the specific genus and/or species^45^. In particular, Bcc and *Mesorhizobium* isolates were highly resistant to most of the tested antibiotics. *Ralstonia, Cupriavidus*, and Bcc isolates showed similar trends in antimicrobial susceptibility. *Sphingomonas sanguinis* isolates were highly sensitive to vancomycin and many antibiotics inhibiting protein synthesis. *Methylobacterium* and *Bradyrhizobium* showed similar drug-resistant trends with the exception of carbenicillin and chloramphenicol. *Staphylococcus epidermidis* was highly sensitive to most tested antibiotics. Many of the tested isolates were sensitive to NaCl concentrations over 1%, except Bcc and *S. epidermidis* (Supplementary Dataset 1).

### Preferred energy source of ISS potable water isolates

**Fig. 3:**
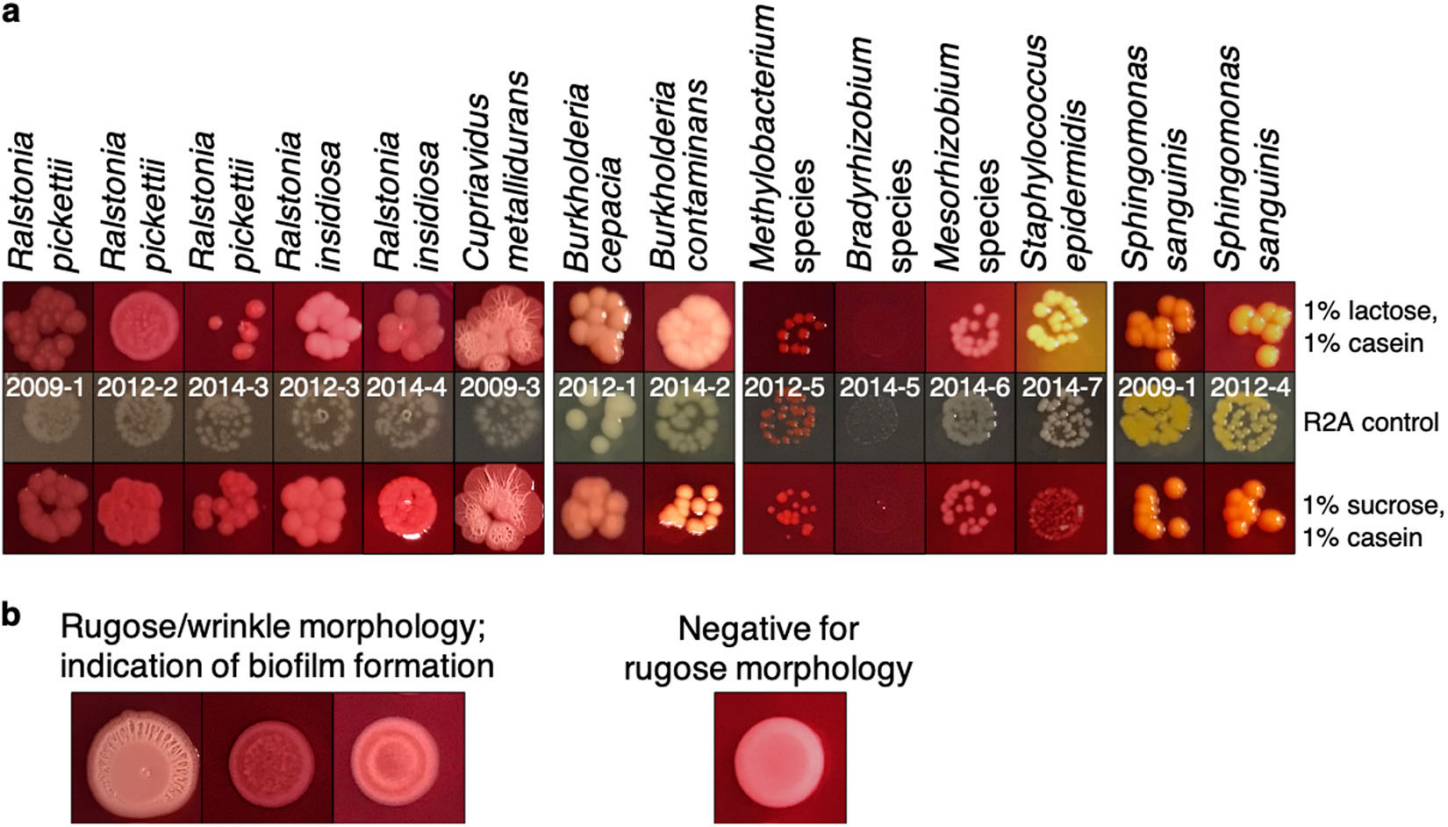
Characteristics of pure cultures of ISS potable water isolates. **a** Core metabolic characteristics and extracellular polysaccharide (EPS) production. The media contains casein and phenol red with 1% lactose or 1% sucrose. Pink or yellow color indicates utilization of carbohydrate or casein amino acids in the presence of both. The colony phenotype on R2A media serves as control. EPS production is indicated with red colonies by 100 μg/ml Congo red. **b** Rugose/wrinkled colony morphology.

We also examined whether carbohydrates or amino acids were the preferred energy source of the ISS isolates. We used agar media containing both casein amino acids and either sucrose or lactose, as well as the pH indicator phenol red. Most species appeared to prefer casein amino acids over either carbohydrate (indicated by the pink color around the colonies) (Fig. 3a). Interestingly, *C. metallidurans* and *R. pickettii* formed a wrinkled/rugose colony morphology, which is a common biofilm phenotype^46^, while *R. insidiosa* produced very smooth colonies (Fig. 3b). We thus decided to further examine biofilm formation of the isolates.

### Biofilm formation and production of extracellular substances

**Fig. 4:**
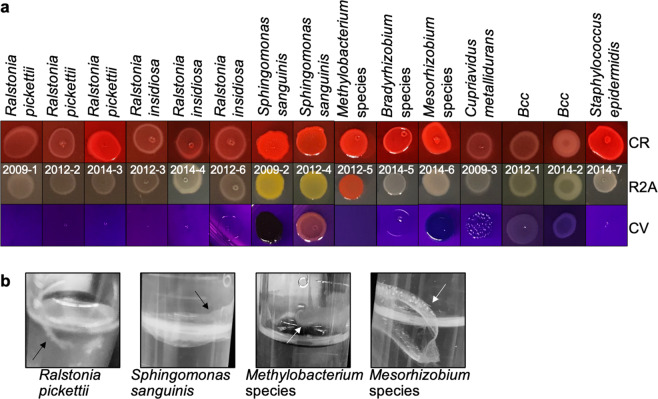
Biofilm-formation tests. **a** The R2A media contains 100 μg/ml Congo red (CR) or 100 μg/ml crystal violet (CV), or no dye addition (control). EPS production (red with CR), biofilm formation (blue–purple on CV). Similar concentrations of bacteria, approximately 10^6^–10^7^ CFU, were plated. **b** The 8-week-old biofilms on air–liquid surface (arrow). Brightness and contrast of pictures were similarly optimized for clarity.

Extracellular polymeric substances (EPS), including extracellular DNA (eDNA), protein, and extracellular polysaccharides, are known to be fundamental components of biofilm growth^47–50^. To detect EPS production by the ISS potable water isolates, we grew bacterial colonies on media containing either Congo red or crystal violet to stain the extracellular substances. Media containing Congo red revealed that strains of *R. pickettii*, *S. sanguinis, Bradyrhizobium spp., Mesorhizobium spp*., and *S. epidermidis* were strong EPS producers with bright-red colonies (Fig. 4a**)**. The crystal violet-containing agar also indicated biofilm formation. We thus further validated biofilm formation using an air–liquid-surface approach for *Methylobacterium, Mesorhizobium, R. pickettii* and *S. sanguinis* (Fig. 4b). All of the tested strains (except *R. insidiosa*) provided phenotypic evidence of biofilm formation with rugose/wrinkle colonies, EPS production, and/or air–liquid-interface biofilms.

### Biofilm ultrastructure, composition, and cellular morphology

**Fig. 5:**
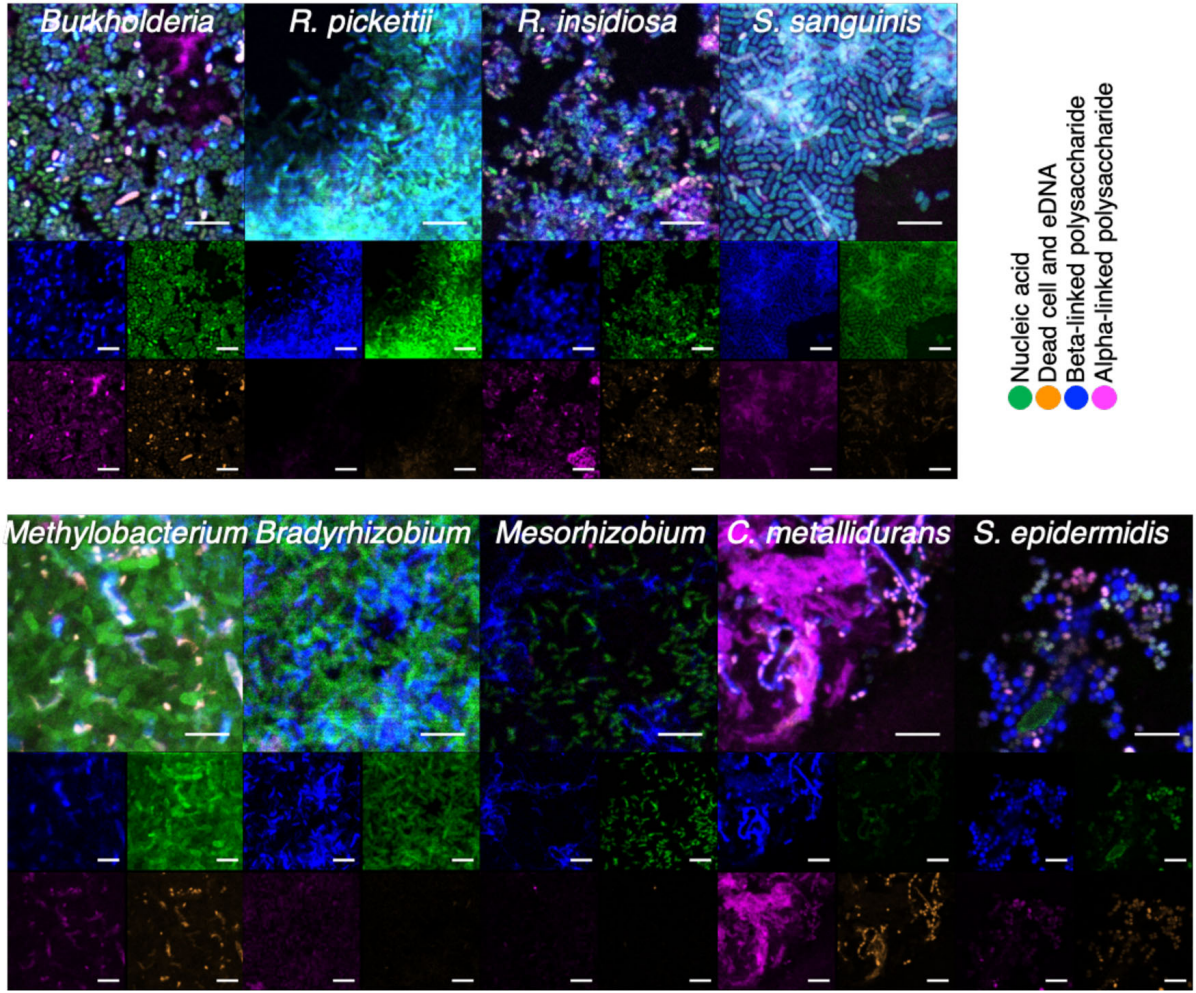
CLSM analysis of static biofilm on glass surfaces formed by ISS potable water isolates. Single-species biofilms were developed statically on glass surface. Cells and EPS compounds were labeled by fluorescent dyes SYTO-9 (green), EthHD-2 (orange), CFW (blue), and ConA-alexa 633 (magenta). Biofilm structures and the composition were observed by CLSM. Scale bars indicate 5 µm.

We used confocal laser scanning microscopy (CLSM) to examine biofilm structures and production of the biofilm-related EPS (Fig. 5, Supplementary Fig. 1). Single-strain biofilms were developed on sterile microscope glass cover slides. Live or dead bacterial cells, EPS compounds, and α- or β-linked polysaccharides were labeled by fluorescent dyes. The dyes used were SYTO-9 (green nucleic acid stain), ethidium homodimer-2 (EthD-2) (orange-dead cell stain), concanavalin A (ConA) (magenta α-linked polysaccharide stain), or calcofluor white (CFW) (blue β-linked polysaccharide stain). Figure 5 shows microscopic imaging of the bacterial spatial organization, biofilm-associated substances, and morphology of biofilms (Fig. 5, Supplementary Fig. 1). The organisms show different biofilm patterns as evidenced by Con A, CFW, and viability staining. In particular, *S. sanguinis*, *Ralstonia*, and *C. metallidurans* formed large clustered biofilms (Fig. 5 and Supplementary Fig. 1). While *Methylobacterium spp*. grew 1–2 logs less in planktonic culture as compared with other species (Supplementary Fig. 2), they formed relatively thick biofilm layers on glass (Supplementary Fig. 1). Interestingly, our data showed that CFW appeared to stain individual bacterial cell surfaces, while ConA appeared to target biofilm matrix components. eDNA was also present in the matrix as evidenced by EthD-2 staining.

### Identification of intraspecies variations and hemolytic *Burkholderia* species

**Fig. 6:**
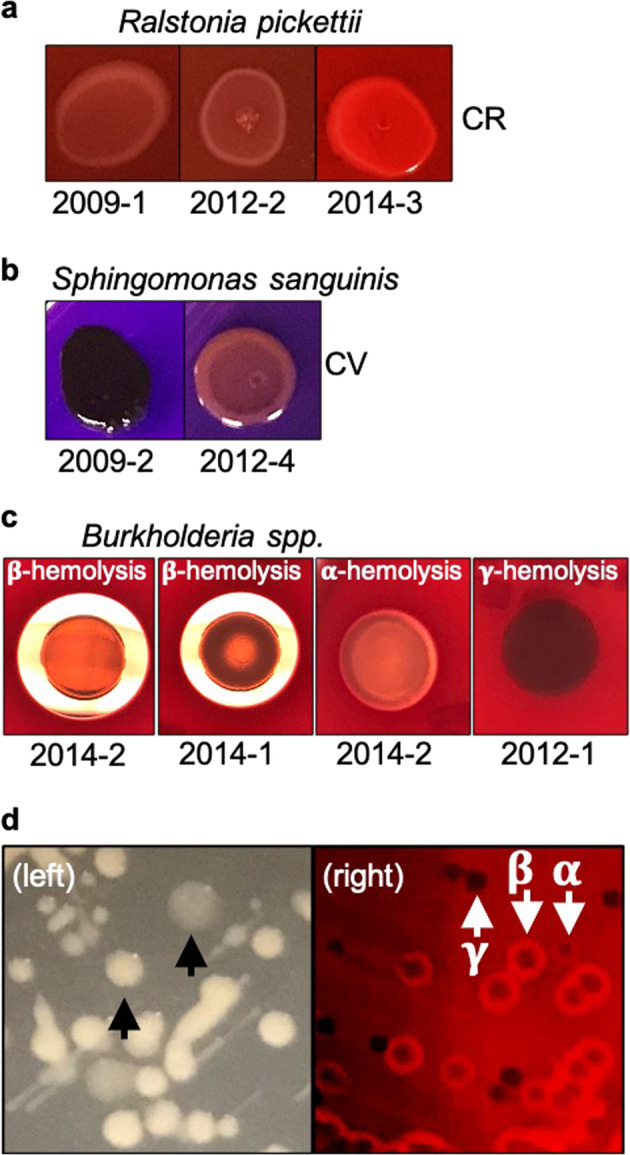
Hemolytic subpopulations of the ISS potable water microbiota and intraspecies variation. **a–c** Phenotypic variations among different isolates of the same species. The number below each image indicates different isolates. a Variations of *R. pickettii* isolates with 100 μg/ml Congo red (CR) staining. **b** Variations of *S. sanguinis* isolates with 100 mg/ml crystal violet (CV) staining. **c** Variation of *Burkholderia* spp. for hemolytic characteristics. Individually grown bacteria from a single colony were spot-dropped on tryptic soy agar with 5% sheep blood and incubated for 24 h. **d** Colony variation of *Burkholderia* spp. for colony morphological phenotypes (left) and hemolytic activity (right). The left picture shows yellow or pale-gray color colonies indicated with arrows. On the right image, the *α*-, *β*-, and *γ*-hemolysis are indicated with arrows demonstrating no-, weak-, and complete lysis of red cells, respectively.

During the course of our studies, we observed that some ISS isolates of the same species, which were recovered in different years, showed evidence of possible intraspecies variation. Specifically, we found variation in the production of extracellular substances among select isolates of *R. pickettii* and *S. sanguinis* (Fig. 6a–b). Visual inspection of the 2014 *R. pickettii* isolates and the 2009 *S. sanguinis* isolates indicated that they may produce more EPS relative to the same species recovered in different years, suggesting intraspecies variation. In addition, we discovered hemolytic activity of select *Burkholderia* isolates. Specifically, the 2014 *Burkholderia* isolates displayed hemolytic activity, while the 2012 isolates did not (Fig. 6c). Surprisingly, our data revealed that the 2014 *Burkholderia* isolates exhibited unstable hemolytic phenotypes and variations in colony morphology (Fig. 6d**right**). Both hemolytic (α and β) and nonhemolytic (γ) colonies were simultaneously found on the same blood agar plate. In addition, both yellow and gray colonies simultaneously appeared on modified R2A (mR2A) agar and gray colonies were visibly more mucoid, suggesting varying mucus/pigment production among individual colonies (Fig. 6d**left**). Moreover, the passaging of a single colony also produced colony variation, suggesting that this phenotype was not due to contamination but instead may result from an unstable phenotype. The different colony types shown in Fig. 6d were indistinguishable from each other based on 16 S rRNA gene sequencing (data provided by NASA), confirming that these morphologically distinct colonies were not contaminants. These differences in colony characteristics might be explained by recent sequencing data showing that the 2012 and 2014 *Burkholderia* ISS potable water isolates are different species^43^. The intraspecies differences in 2014 *Burkholderia* colony morphology and pigment were not observed using commercially available R2A, TSA, or LB agar media. Surprisingly, the 2014 *Burkholderia* isolates that exhibited β-hemolysis also tested positive for beta-galactosidase, urease, and D-trehalose, which are uncommon for Bcc strains (Supplementary Table 3).

### Interspecies interactions

**Fig. 7:**
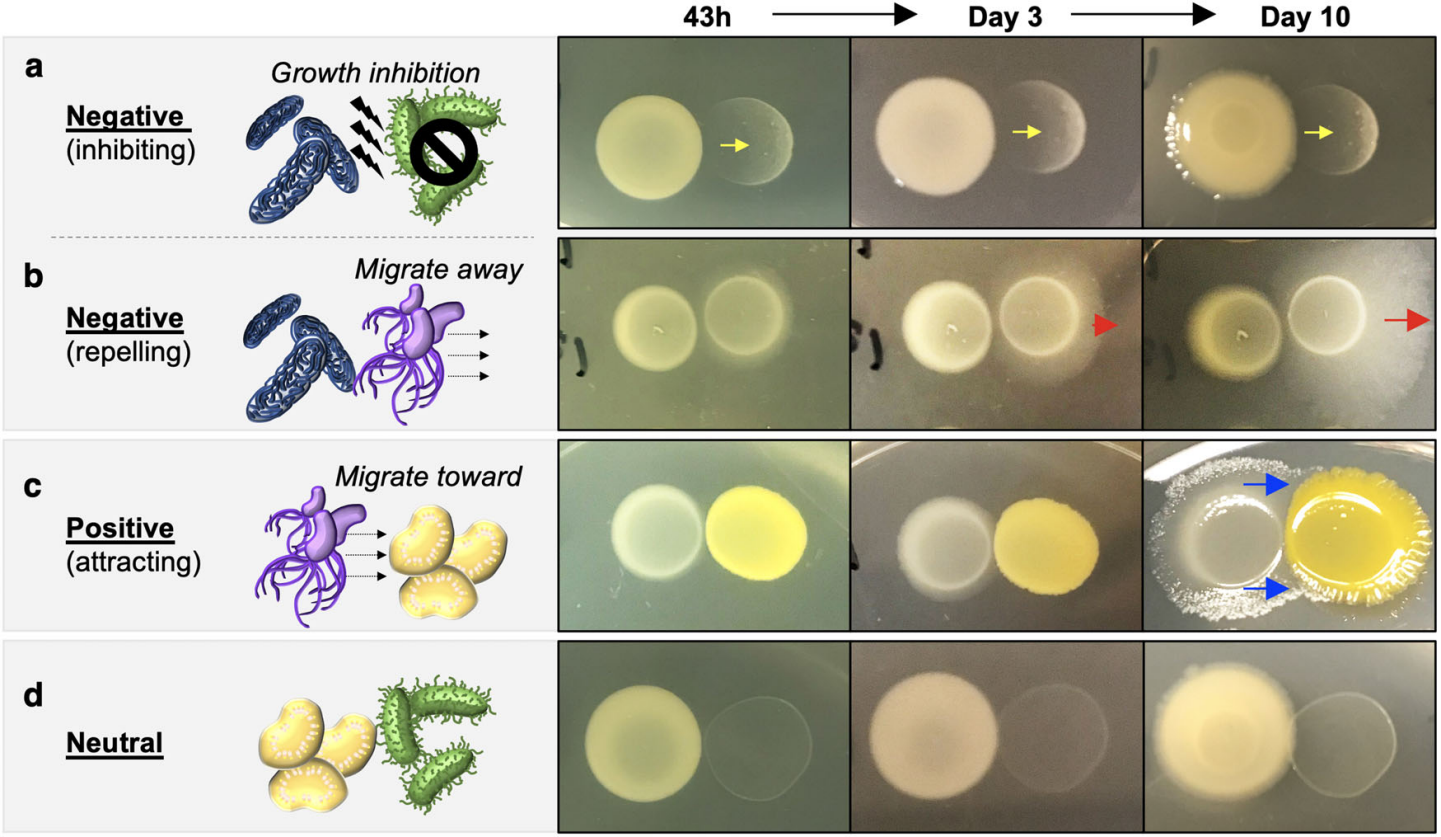
Types of interspecies interactions and their progressive changes over time. Three distinct interspecies interactions were observed in this study. Interaction criteria were based on migration of the cultures during growth in close proximity. Rows **a** and **b** show negative interactions, where one organism killed/inhibited (**a**, yellow arrows) or grew away from the other strain (**b**, red arrows). Row **c** shows positive interaction, where one strain grew out toward and in some cases surrounded the other (blue arrows). Row **d** shows neutral interaction, where no visible sign of interaction was observed between the organisms. The colonies on the left and right in each panel are respectively: **a**, 2014 *Burkholderia spp*. and 2014 *Staphylococcus spp*.; **b**, 2012 *Burkholderia spp*. and 2012 *Ralstonia spp*.; **c**, 2012 *Ralstonia spp*. and 2012 *Sphingomonas spp*.; **d**, 2014 *Burkholderia spp*. and 2014 *Bradyrhizobium spp*.

To better understand microbial relationships between different species that may occur in the ISS potable water system, we examined interactions between these species. Individual strains were spotted onto R2A agar directly adjacent to different species to assess the interactions between every possible combination of paired isolates (Supplementary Fig. 3). Interspecies interactions between the paired isolates over a progressive time course were rated as negative, positive, or neutral, based on migration of the cultures during growth in close proximity (Fig. 7). Neutral relationships were defined when two strains did not show a visible sign of interaction. Positive relationships were defined when one strain grew out toward and in some cases surrounded the other strain (Fig. 7c). We found two types of negative behaviors. For example, *Methylobacterium* and *S. epidermidis* were either killed or their growth inhibited by *Burkholderia* (Fig. 7a), while *R. insidiosa* grew away from *Burkholderia* (Fig. 7b). *Bradyrhizobium* and *Mesorhizobium*, which are both slow-growing bacteria and thus could be potentially less competitive relative to faster-growing species, displayed no growth defect when interacting with most strains, thus showing neutral relationships (Fig. 7d).

**Fig. 8:**
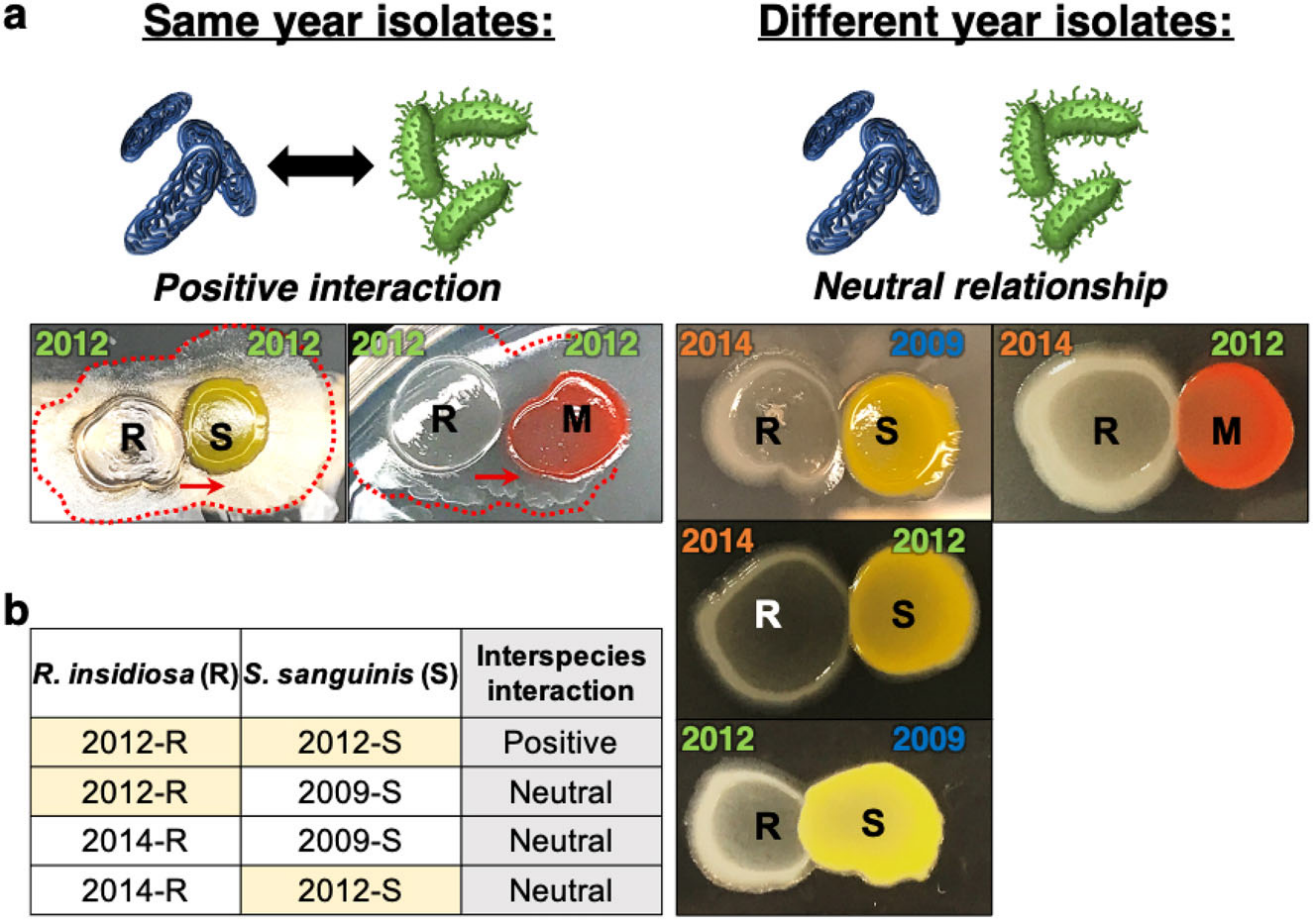
Interspecies interactions may be influenced by the year of isolation. **a** Distinct relationships observed between strains isolated in the same year but not in different years. R, S, or M indicate *R. insidiosa, S. sanguinis*, or *Methylobacterium* spp., respectively. Red dotted lines highlight where *Ralstonia spp*. have grown out toward and surround the other species. **b** Correlation between the isolation year and interactions between different species exhibiting positive or neutral relationships. The years isolated are indicated above colonies, shown in the same color.

We also observed a correlation between isolation year and the type of interspecies interaction between *R. insidiosa* and either *S. sanguinis* or *Methylobacterium*. For these strains in our study, species isolated in the same year exhibited positive relationships, while neutral relationships were observed when species from different years were cocultured (Fig. 8a–b).

### Mixed-species colony biofilms

We performed mixed-species colony biofilm assays to examine microbial multispecies behavior by combining different strains isolated in the same year and then plating these mixed cultures as a single colony. Strains were first individually cultured in liquid media, then equal ratios of either (1) two species from the same year, or (2) all isolates from each year (2009, 2012, or 2014) were mixed and spotted as a single colony onto R2A agar to compare the characteristics of colony biofilms. The morphologies of these mixed-species colonies were monitored for 10 days. Distinct phenotypes were observed for each mixed-species colony that resembled the dominant species in the mixture, but still differed from the morphology of the individual strains (Supplementary Fig. 4). *S. sanguinis*, *C. metallidurans*, *R. insidiosa*, or *Burkholderia* tended to exhibit relatively dominant phenotypes as evidenced by colony color or morphology. *Methylobacterium* spp., which previously showed growth inhibition when spotted next to *a Burkholderia* colony (Supplementary Fig. 3), did not show visible signs of growth in the colony when directly mixed with *Burkholderia*.

## Discussion

Microbial interactions within natural and artificial environments are typically investigated over a short period of time (hours to days). Longer-term interactions may only become evident during repeated observations of the same environment. Microbial observatories have been established in a number of environments^51–53^. These studies show how long-term changes may occur in a community due to alterations of environmental factors or the introduction of new species. In the current study, we used the ISS water-recovery system as a microbial observatory. Unique aspects of this environment include microgravity and the limited opportunities for the introduction of new microorganisms via crew exchanges and resupply missions^8,54^. Frequent microbial sampling is performed to mitigate risks to crew health and system performance^10,11^. While identification of isolates can provide useful information, phenotypic analysis enables an increased understanding of long-term effects of the spaceflight environment on microbial physiology and functional species’ interactions. In this study, we characterized antimicrobial susceptibility, single- and multispecies biofilm formation, EPS production, hemolytic subpopulations, preferred energy source, and multispecies interactions of sixteen ISS potable water isolates. Our analysis using archived ISS isolates showed that *Ralstonia and Burkholderia*, known biofilm formers, were the major bacterial genera between 2009 and 2016.

Microbes interact with their neighbors in mixed communities and compete for space and resources in diverse environments^55–59^. Polymicrobial interactions between water isolates have been shown to affect biofilm functional characteristics, including drug resistance and morphology^60–64^. However, none of these studies were longitudinal in nature. Of particular relevance to our study, Thompson et al.^64^ recently conducted elegant experiments using six ISS potable water microbial isolates (including some of the same species used in our study) and showed the contribution of individual community members to the robustness of polymicrobial biofilm formation. Interestingly, this work found that some bacterial community members were necessary for polymicrobial biofilm formation and that predation by phage or predatory bacteria did not selectively remove specific bacterial community members. While microbial interactions may appear to be stable in short-term studies, this may not be true for longer-duration interactions^65–67^. Such microbial dynamics are critical to better understand the types of interactions and functional relationships that may occur in environments such as the ISS potable water microbial community over time. These studies highlight the importance of investigating the effects of long-term polymicrobial interactions in many different environments. Indeed, complex relationships between cohabiting species and a loss of antagonism over time have been frequently documented in longitudinal studies of pulmonary isolates from cystic fibrosis (CF) patients^68–71^. A recent longitudinal study using *Pseudomonas aeruginosa* clinical isolates from CF patients suggested that the long-term development of metabolic divergence contributed to cooperative interspecies interactions that evolved over decades^72,73^.

**Fig. 9:**
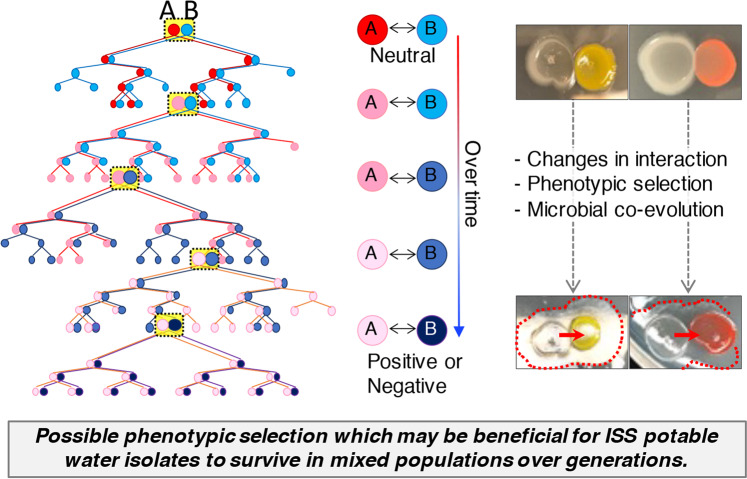
Hypothetical microbial coevolution model associated with interspecies interactions of ISS water isolates indicates potential formation of new relationships over time. A and B indicate different bacterial genera.

Using bacterial isolates recovered over time from the same ISS potable water system, we observed positive, negative, or neutral interspecies relationships with temporal dependent interspecies interactions observed between certain strains. As the section of the water system containing these isolates is downstream of a 0.2-micron water filter, introduction of outside contamination into the system would be highly unlikely^8,15,74^. Thus, the isolates collected at any given sampling timepoint may reflect bacteria that have coexisted for extended time periods. As these bacterial communities will have changed over time, isolates collected during a given year (e.g., 2009) may interact differently with bacteria collected from other years (e.g., 2012, 2014). Our data corroborate this possibility. Based on our findings, we propose a hypothetical microbial coevolutionary model to explain the observed interspecies interactions that might have developed during spaceflight (Fig. 9). We recognize that our results may be explained by other possibilities, including (a) the limited number of strains tested in this study, (b) potential phenotypic changes that might result from culturing the isolates in our laboratory as opposed to microgravity conditions in spaceflight, (c) different nutrient availability between the ISS water system and conditions used in this study, (d) strains isolated in later years might have been introduced via contamination into the ISS water system, and (e) species compared over different time periods were represented by different strains. Regardless, data from this study provide a foundation from which to advance our understanding of multispecies microbial communities and their functional interactions in the ISS potable water system over time, which could impact crew health and system performance.

It is worth noting that studies have reported reduced immune function in astronauts during spaceflight, which when coupled with alterations in virulence, biofilm formation and antibiotic resistance observed in some microorganisms during spaceflight, suggests an increased risk for infectious disease^28,29,31,34,35,75–78^. Our observation of hemolytic *Burkholderia* subpopulations that were multidrug resistant in ISS potable water samples confirms previous work by O’Rourke et al.^43^ and suggests an additional risk for opportunistic infections. We expanded on these previous findings^43^ by profiling the type of hemolysis (*α*, *β*, *γ*) in the *Burkholderia* isolates and discovered unstable hemolytic phenotypes, as well as variations in colony morphology and pigmentation. Interestingly, there was no clear association between hemolysis, mucoidy, or colony pigmentation. These same three phenotypes were unstable during our testing and produced the observed unpredictable colony variations during repeated passage on agar plates. To our knowledge, this is the first report that ISS Bcc isolates exhibit unstable hemolysis, mucoidy, and pigmentation phenotypes. These unstable pathogenic characteristics also occur in several organisms on Earth^79–82^. Notably, studies using microbial communities isolated from terrestrial water systems have shown increased resistance to antimicrobial agents and enhanced biofilm formation^61^. In addition, studies using microbial communities isolated from spaceflight water systems have shown increased biofilm formation and bacterial predation in multispecies biofilms^31,61,64^. Should the same phenotypic instability occur in spaceflight, it could present a challenge for detection and pose a risk for astronaut health. Further investigation is in progress to understand the role of microbial community interactions in antibiotic resistance, the virulence of select ISS potable water isolates, and potential underlying mechanisms.

Our data also revealed distinct patterns of biofilm formation by the ISS potable water isolates that may be representative of the different species tested in this study. One potential explanation of the diverse biofilm patterns observed may be the different extracellular matrix components, as revealed by ConA and CFW staining, which stain α- and β-linked polysaccharides, respectively. We also observed that EthD-2, which does not penetrate the membrane of living cells, was observed at unexpectedly high levels in *S. sanguinis, Ralstonia spp., C. metallidurans*, *Methylobacterium*, and *S. epidermidis*. The increased EthD-2 signal in these cells may reflect eDNA in the biofilms, as has been previously shown with similar species^47,83–85^. We plan to conduct future studies to identify the mechanisms involved in these bacterial interactions and biofilm formation.

Our longitudinal study provides a comprehensive phenotypic investigation of ISS potable water microbial isolates, focusing on functional characteristics of the different species alone or as multispecies consortia. As joint molecular and phenotypic analyses can provide a more comprehensive understanding of polymicrobial interactions and function than either approach alone, we are currently investigating molecular mechanisms that may explain some of the functional phenotypes that were observed for several of the ISS isolates in this study. By profiling the functional phenotypes of ISS isolates over a series of kinetic timepoints, the results of this study offer potential insight into how polymicrobial isolates may contribute to biofouling of onboard life-support systems and impact human health in space and on Earth.

## Methods

### Strains and culture conditions

Strains were obtained from NASA and isolates were identified by 16 S rRNA gene sequencing and/or a biochemical identification using a VITEK system (BioMerieux) by the NASA Johnson Space Center (JSC). Lists of ISS potable water isolates archived by NASA from the US water system between 2009 and 2015 were obtained online^44^. Diversity assessments using these publicly available data were performed as described in the text. Potable water isolates used in this study from early (2009), middle (2012), and late (2014) years were provided by NASA JSC on commercial R2A media. Strains were cultured at 28–29 °C in a humidified chamber for 1–10 days on the following solid media: TSA (MP biomedicals, 091010717; Difco 214530), R2A agar (Becton, Dickinson, 218263), or mR2A agar (modified R2A agar; Teknova, R0005; Difco 214530). Prior to each experiment (for all strains), single colonies were isolated on R2A agar (1.5% agar) or mR2A agar (1.3% agar) from frozen stocks, and individual strains were cultured in R2A broth (Teknova, R0005) at room temperature to stationary phase of growth. The growth kinetics of each strain were determined at the beginning of the study to confirm that experiments were all performed at the same phase of growth.

### Antimicrobial susceptibility testing (AST)

Strains were individually cultured to stationary phase in R2A broth, and 10-fold serial dilutions were prepared. We chose to test a range of antibiotic concentrations in our study based on literature searches, which included (a) concentrations commonly used for the same or similar organisms, including minimum inhibitory concentrations, and (b) reports that spaceflight can profoundly alter the antibiotic susceptibility of some bacteria^86,87^. Diluted and undiluted samples were plated on mR2A plates containing antibiotic(s) or NaCl at specified concentrations and incubated at 28–29 °C in a humidified chamber. The AST plates were monitored for approximately one week and bacterial survival was scored by the dilution showing microbial growth.

Tenfold dilutions of a pure culture were plated on R2A agar media containing specific concentrations of antimicrobial compounds. Antimicrobial susceptibility was scored by testing the log decrease in bacterial survival, from 0 (no to <1 log-fold decrease) to 7 (7 or more log-fold decrease) (Supplementary Fig. 5a). The antimicrobial susceptibility was evaluated using CLSI (Clinical and Laboratory Standards Institute) guidelines (Supplementary Fig. 5b)^45^.

### Energy-metabolism tests

The casein-PR agar media used for metabolism testing contained 1% Casein (Sigma-Aldrich, 22090), 0.5% NaCl (S3014), 0.15% Bacto Yeast extract (BD, 212750), 0.008% Phenol red (PR, Sigma-Aldrich, P4758), and 1.3% agar. For additional supplements, 1% sucrose (Sigma-Aldrich, S7903) or 1% lactose (Sigma, 61345) was added to the casein-PR media. Experiments were performed using the same methods for the AST studies.

### Biofilm tests

mR2A plates containing Congo red (Sigma–Aldrich, C6767) 100 µg/ml, or crystal violet 100 µg/ml, were prepared. Experiments were performed using the same methods for the AST studies. For air–liquid-surface biofilms, individual strains were inoculated in 5 mL of R2A broth, standing and development of biofilms were monitored for eight weeks. Photos were taken at eight weeks post inoculation.

### Confocal laser scanning microscopy (CLSM) and sample preparations

Biofilms were developed on sterile glass cover slips in broth cultures of each individual strain for 24 h at RT. Cover slips containing pure culture biofilms were subsequently washed with sterile Dulbecco’s phosphate-buffered saline (DPBS) to remove planktonic bacteria and fixed with 4% paraformaldehyde (PFA, Electron Microscopy Sciences) for 20 mins, following gentle washing with cold DPBS twice. In a limited-light environment, the samples were stained with SYTO-9 green fluorescent stain (10 mg/mL, nucleic acid stain, Thermo Fisher Scientific S34854) for 15 min, Concanavalin A conjugated with Alexa Fluor™ 633 (500 mg/L, α-polysaccharide stain), Ethidium Homodimer-2 (EthD2, 1 mg/mL, dead-cell stain, Thermo Fisher Scientific E3599), or Fluorescent Brightener 28/Calcofluor White (0.7 mg/mL, β-polysaccharide stain, Sigma F3543) for five minutes.

Biofilms were imaged using a Leica TCS SP8 LSCM with sequential scanning. Images were processed using Leica LAS software. Three to six stacks of horizontal plane images (1024 × 1024 pixels) with a z-step of 3 μm (the distance between the images) were acquired for each sample from two or more randomly chosen areas. Two independent experiments were performed. Fluorescence images were acquired with excitation at 405 nm (1.57%), 488 nm (4.98%), 514 nm (10.1%), or 633 nm (3.1%); emission filter DAPI (420–460 nm), FITC (498–545 nm), mCherry (565–610 nm), or Alexa-644 (648–700 nm) and gain 540.2 V (PMT), 60% (HyD), 65% (HyD), or 60% (HyD). Image scanning was carried out by sequential scanning with seq-1 (405-nm laser, pinhole 0.31), seq-2 (488 nm and 633 nm, pinhole 0.32), and seq-3 (514 nm, pinhole 0.31).

### Hemolysis test

Experiments were performed using the same methods for the AST studies, but instead used blood agar (BA; TSA with sheep blood, Fisher R01200). The plates were incubated and monitored for 15–72 h.

### Interspecies and mixed-species interaction testing

Individual strains were grown in R2A broth as described above. Cells were centrifuged at 7000 r.p.m. for 15 mins and pellets were resuspended in fresh R2A broth to concentrate by 10 times. To assess interactions between designated strains, 10-µl samples of each isolate were dropped onto R2A agar (1.5%) at the same time at a distance close enough to allow for interaction but far enough away to avoid fusion of the droplets. Pairwise combinations of every strain in this study were examined. For mixed-species assays, samples were prepared in the same way to test the interaction between two strains. Equal volumes of the 10X- concentrated strains were mixed and 10 µl of these mixtures were drop-applied on R2A agar (1.5%). Different strains recovered from the same year were tested as one set to study mixtures of two species or all species. Incubation, monitoring, and recording were conducted as described above.

### Data collection, data analysis, and statistical analysis

For most assays (except hemolysis and CLSM), microbial growth on plates was monitored over seven days by taking photos and recording observations every 1–2 days, mostly due to the varying growth speeds for different strains. All assays presented in this paper were repeated at least in triplicate. All statistical analyses were performed using Graphpad Prism 7 or Microsoft Excel. Mean value and error bars (standard error of the mean) are indicated.

### Reporting summary

Further information on research design is available in the Nature Research Reporting Summary linked to this article.

## Supplementary information

Supplementary Information

Supplementary Data 1

Reporting Summary

## Data Availability

Datasets that support the findings of this study are available from the corresponding author on reasonable request.
