# Supplementary material for: Longitudinal characterization of multispecies microbial populations recovered from spaceflight potable water
Source: NPJ Biofilms Microbiomes. 2021 Sep 6;7:70. doi: 10.1038/s41522-021-00240-5 (PMC8421509; doi:10.1038/s41522-021-00240-5)
Supplement: Supplementary file 1 — Supplementary Information [file 41522_2021_240_MOESM1_ESM.pdf]

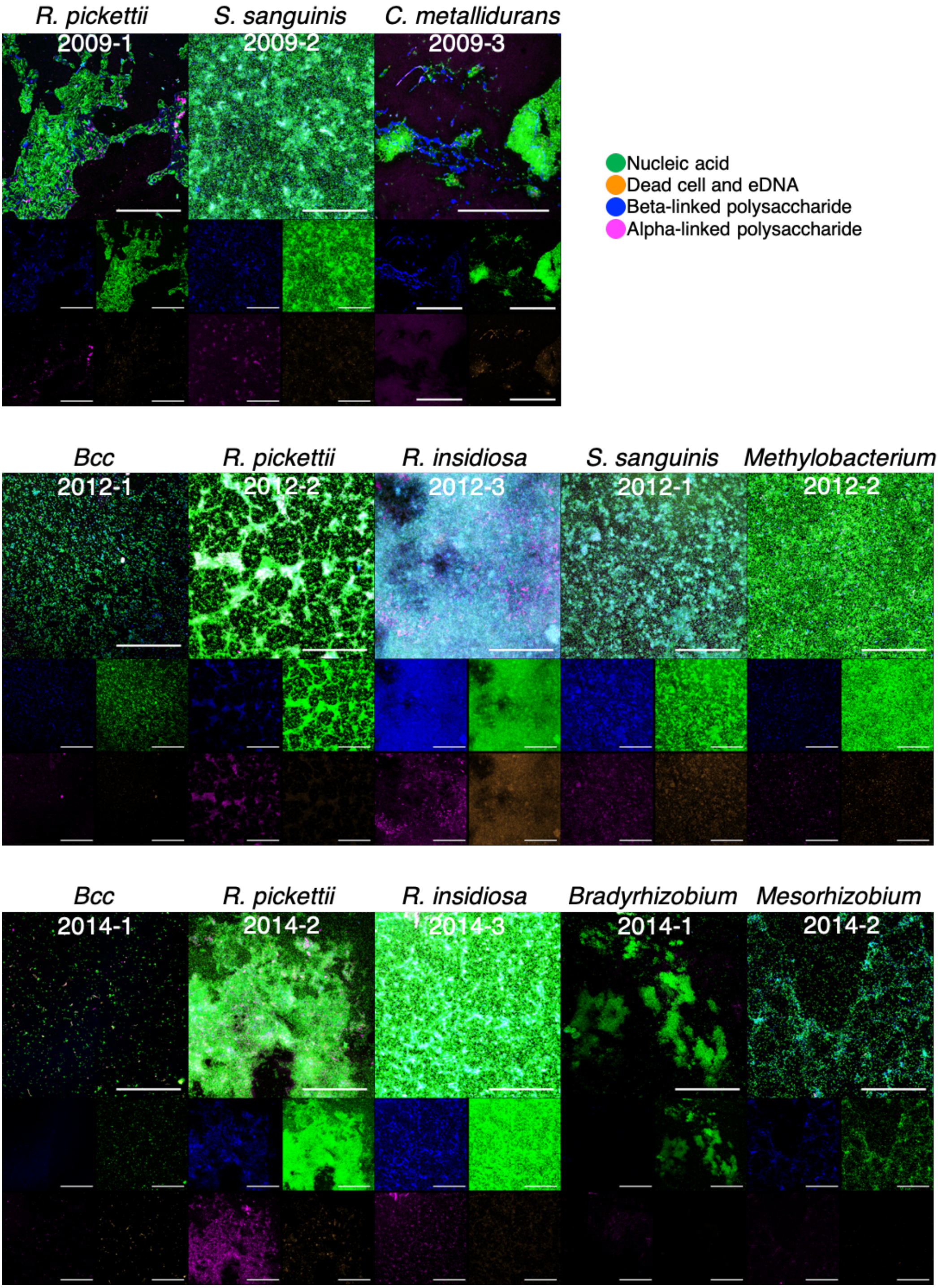

Supplementary Figure. 1. CLSM analysis of static biofilm on glass surface formed by ISS potable water isolates. Single species biofilms were developed on glass surface statically. Cells and EPS compounds were labeled by fluorescent dyes of SYTO-9 (green), EthHD-2 (orange), CFW (blue channel), and ConA-alexa 633 (magenta) and biofilm structures and composition were observed by CLSM. The details are given in the materials and methods section. Scale bars indicate 100 μm. The number below each image indicates different isolates.

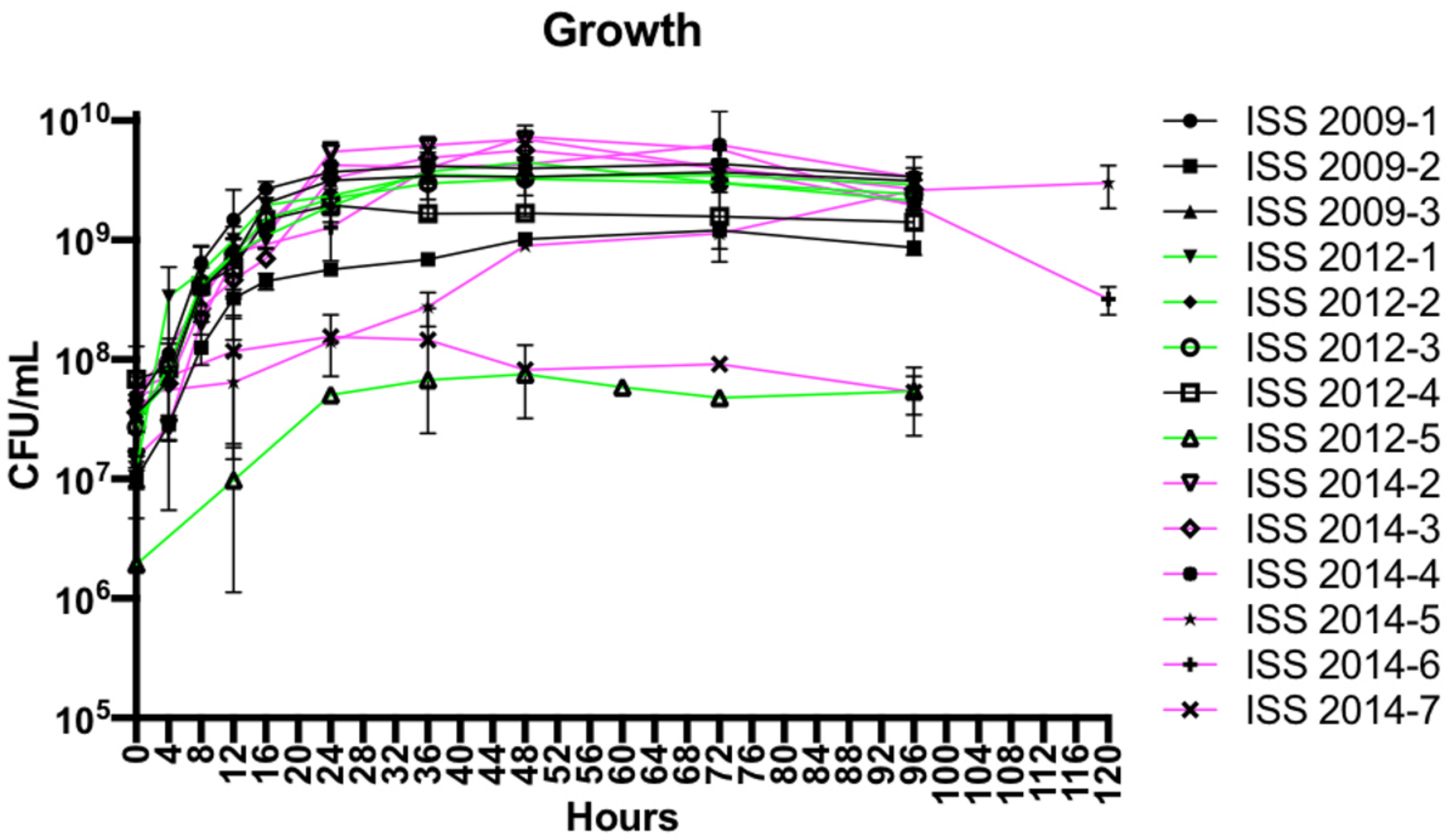

Supplementary Figure 2. Growth curve of individual ISS potable water isolates. Individual strains were cultured in 5mL Reasoner's 2A (R2A) liquid medium in sterile polypropylene snap cap tubes at room temperature and shaken at 130 rpm. The growth was monitored for 96 or 120 hours by determining the CFU/mL on nonselective R2A medium. Experiments were done a minimum of 3 times. The mean value and standard deviation error bars are shown for each time point.

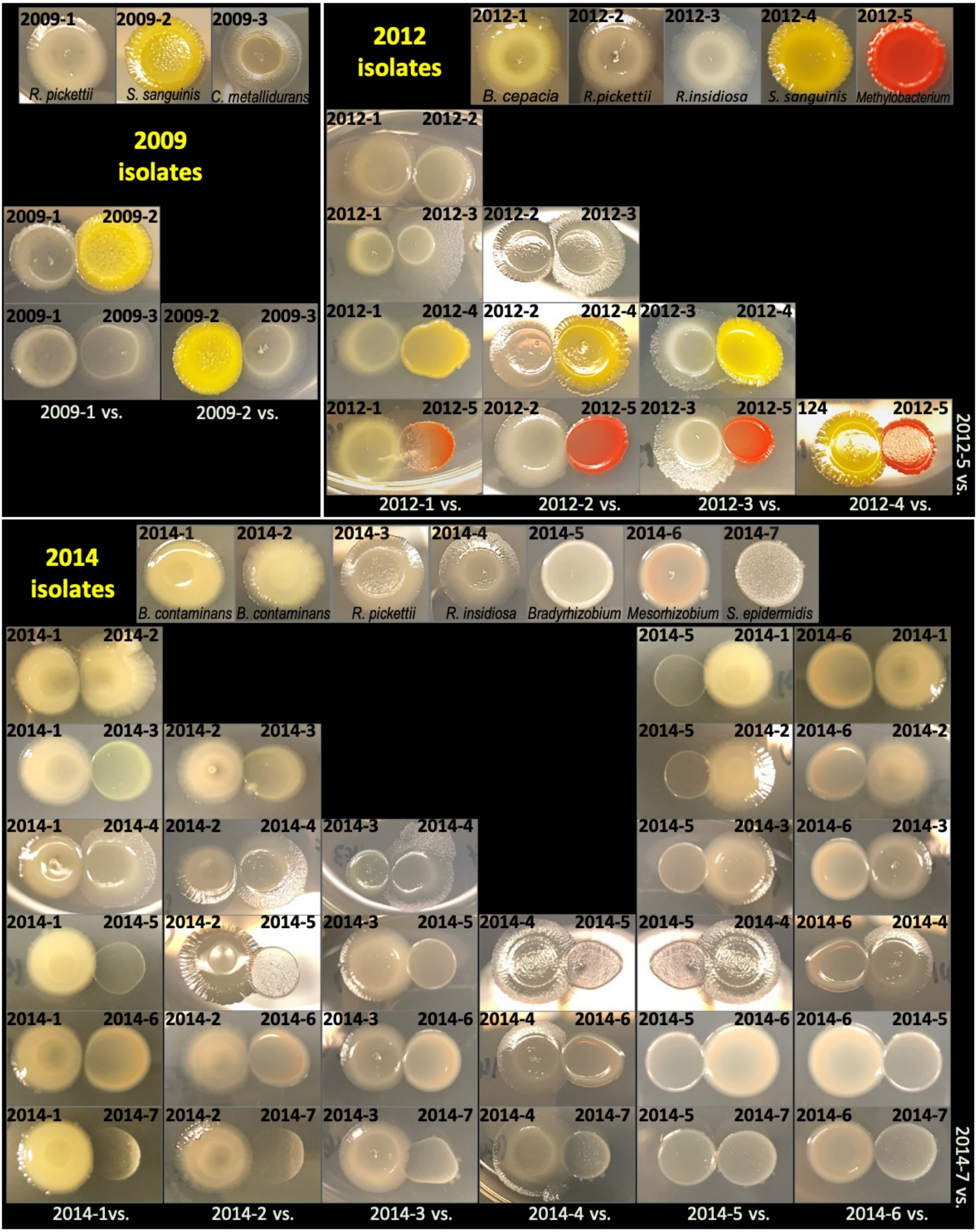

Supplementary Fig. 3. Interspecies interaction between ISS potable water isolates. High density colony morphology of individual strains and interactive colony morphological phenotypes with every possible combination within the same year group. The interspecies interaction was monitored for about 10 days. The assay was repeated 2-3 times.

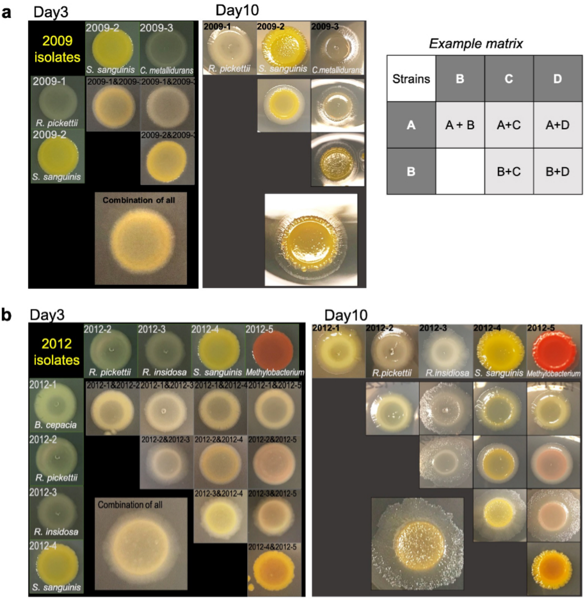

Example matrix

Strains	B	C	D
A	A + B	A+C	A+D
B		B+C	B+D

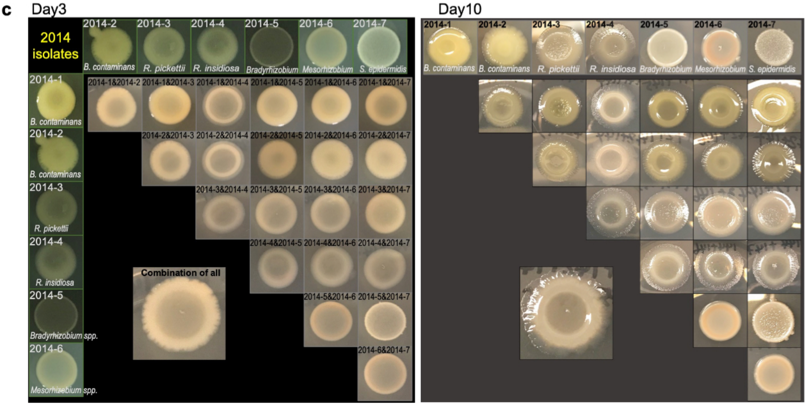

Supplementary Figure 4. Mixed-species colony biofilms. Colony biofilm morphologies of pure and mixed-species. Isolates shown in the top row were mixed with those shown in the left column, as shown in the example matrix.
a 2009 isolates,
b 2012 isolates,
c 2014 isolates.
See **Table 1** for strain identification.

a

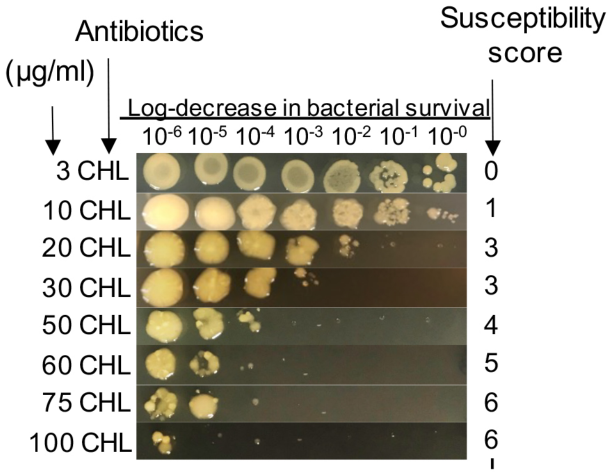

b

Susceptibility score	CLSI determination	Decrease in CFU (approximately)
0	Resistant (R)	10^0
1	Resistant (R)	10^1
2	Resistant (R)	10^2
3	Intermediate (I)	10^3
4	Intermediate (I)	10^4
5	Intermediate (I)	10^5
6	Susceptible (S)	10^6
7	Susceptible (S)	10^7

Supplementary Figure. 5. Antimicrobial resistance of pure cultures of ISS potable water isolates. a Antimicrobial susceptibility testing and scoring. CHL indicates chloramphenicol. **b** The susceptibility scoring and CLSI determination.

Supplemental Table 1. Microbial isolates from US water system in ISS listed in the NASA archive. The number of different genera does not include the unidentified Gram negative rods.

Microbial Identification	Domain	Phylum	Class	Order	Family	The number of isolates
Acinetobacter spp.	Bacteria	Proteobacteria	Gammaproteobacteria	Pseudomonadales	Moraxellaceae	1
Burkholderia spp.	Bacteria	Proteobacteria	Betaproteobacteria	Burkholderiales	Burkholderiaceae	24
Bradyrhizobium spp.	Bacteria	Proteobacteria	Alphaproteobacteria	Rhizobiales	Bradyrhizobiaceae	1
Chitinophaga spp.	Bacteria	Bacteroidetes	Sphingobacteria	Sphingobacteriales	Chitinophagaceae	2
Chryseobacterium gleum	Bacteria	Bacteroidetes	Flavobacteria	Flavobacteriales	Flavobacteriaceae	2
Cryptococcus laurentii	Fungi	Basidiomycota	Tremellomycetes	Tremellales	Tremellaceae	3
Cupriavidus metallidurans	Bacteria	Proteobacteria	Betaproteobacteria	Burkholderiales	Burkholderiaceae	4
Curvibacter spp.	Bacteria	Proteobacteria	Betaproteobacteria	Burkholderiales	Comamonadaceae	1
Leifsonia spp.	Bacteria	Actinobacteria	Actinobacteria	Actinomycetales	Microbacteriaceae	1
Mesorhizobium spp.	Bacteria	Proteobacteria	Alphaproteobacteria	Rhizobiales	Phyllobacteriaceae	10
Methylobacterium spp.	Bacteria	Proteobacteria	Alphaproteobacteria	Rhizobiales	Methylobacteriaceae	4
Pelomonas spp.	Bacteria	Proteobacteria	Betaproteobacteria	Burkholderiales	Comamonadaceae	2
Phyllobacterium spp.	Bacteria	Proteobacteria	Alphaproteobacteria	Rhizobiales	Phyllobacteriaceae	1
Ralstonia spp.	Bacteria	Proteobacteria	Betaproteobacteria	Burkholderiales	Burkholderiaceae	43
Sphingobium yanoikuyae	Bacteria	Proteobacteria	Alphaproteobacteria	Sphingomonadales	Sphingomonadaceae	1
Sphingomonas spp.	Bacteria	Proteobacteria	Alphaproteobacteria	Sphingomonadales	Sphingomonadaceae	8
Staphylococcus epidermidis	Bacteria	Firmicutes	Bacilli	Bacillales	Staphylococcaceae	4
Unidentified Gram-negative rod						5
The No. of of different species						17
The total No. of ISS water isolates						117

Supplementary Table 2. ISS water isolates from 2009 to 2015

	**2009**	**2010**	**2011**	**2012**	**2013**	**2014**	**2015**
No. of spp. (a)	3	5	8	8	12	8	3
No. of isolates (b)	10	22	17	16	18	22	8
**Diversity score (a/b x100)**	30.00	22.73	47.06	50.00	66.67	36.36	37.50
*Ralstonia (**R**) spp. (%)*	80.00	40.91	29.41	31.25	33.33	13.64	87.50
*Burkholderia (**B**) spp. (%)*	0.00	40.91	17.65	31.25	5.56	27.27	0.00
Sphingomonas spp. (%)	10.00	9.09	11.76	6.25	11.11	0.00	0.00
Methylobacterium spp. (%)	0.00	0.00	11.76	12.50	0.00	0.00	0.00
***R + B* (%)**	80.00	81.82	47.06	62.50	38.89	40.91	87.50

Supplementary Table 3. Metabolic profiling of the 2014 beta-hemolytic Bcc strain. Bacterial colonies were tested for biochemical characteristics using a VITEK system for Gram-negative bacteria.

Mnemonic	Test	Results
APPA	Ala-Phe-Pro-ARYLAMIDASE	-
ADO	ADONITOL	+
PyrA	L-Pyrrolydonyl-ARYLAMIDASE	-
lARL	L-ARABITOL	+
dCEL	D-CELLOBIOSE	+
BGAL	BETA-GALACTOSIDASE	+
H2S	H2S PRODUCTION	-
BNAG	BETA-N-ACETYL-GLUCOSAMINIDASE	+
AGLTp	Glutamyl Arylamidase pNA	-
dGLU	D-GLUCOSE	+
GGT	GAMMA-GLUTAMYL-TRANSFERASE	+
OFF	FERMENTATION/ GLUCOSE	-
BGLU	BETA-GLUCOSIDASE	-
dMAL	D-MALTOSE	+
dMAN	D-MANNITOL	+
dMNE	D-MANNOSE	+
BXYL	BETA-XYLOSIDASE	-
BAlap	BETA-Alanine arylamidase pNA	+
ProA	L-Proline ARYLAMIDASE	+
LIP	LIPASE	-
PLE	PALATINOSE	-
TyrA	Tyrosine ARYLAMIDASE	+
URE	UREASE	+
dSOR	D-SORBITOL	+
SAC	SACCHAROSE/SUCROSE	+
dTAG	D-TAGATOSE	-
dTRE	D-TREHALOSE	+
CIT	CITRATE (SODIUM)	+
MNT	MALONATE	+
5KG	5-KETO-D-GLUCONATE	-
lLATk	L-LACTATE alkalinisation	+
AGLU	ALPHA-GLUCOSIDASE	-
SUCT	SUCCINATE alkalinisation	+
NAGA	Beta-N-ACETYL-GALACTOSAMINIDASE	+
AGAL	ALPHA-GALACTOSIDASE	-
PHOS	PHOSPHATASE	+
GlyA	Glycine ARYLAMIDASE	-
ODC	ORNITHINE DECARBOXYLASE	-

LDC	LYSINE DECARBOXYLASE	-
0DEC	DECARBOXYLASE BASE	
lHlSa	L-HISTIDINE assimilation	-
CMT	COURMARATE	+
BGUR	BETA-GLUCORONIDASE	-
O129R	O/129 RESISTANCE (comp.vibrio.)	-
GGAA	Glu-Gly-Arg-ARYLAMIDASE	-
lMLTa	L-MALATE assimilation	-
ELLM	ELLMAN	-
lLATa	L-LACTATE assimilation	+
